# The Effect of Chronic Exercise on Energy and Fatigue States: A Systematic Review and Meta-Analysis of Randomized Trials

**DOI:** 10.3389/fpsyg.2022.907637

**Published:** 2022-06-03

**Authors:** Carly L. A. Wender, Mika Manninen, Patrick J. O’Connor

**Affiliations:** ^1^Center for Traumatic Brain Injury Research, Kessler Foundation, East Hanover, NJ, United States; ^2^Department of Physical Medicine and Rehabilitation, Rutgers New Jersey Medical School, Newark, NJ, United States; ^3^School of Health and Human Performance, Dublin City University, Dublin, Ireland; ^4^Exercise Psychology Laboratory, Department of Kinesiology, College of Education, University of Georgia, Athens, GA, United States

**Keywords:** energy, emotions, exercise, exercise training, fatigue, meta-analysis, physical activity, vitality

## Abstract

In this meta-analysis, we synthesized the results of randomized controlled trials of different exercise training interventions on participants’ feelings of fatigue, energy, and vitality. The search of studies was conducted using six databases as well as several other supplementary search strategies available before December 2021. The initial search generated over 3,600 articles with 81 studies (7,050 participants) and 172 effects meeting the inclusion criteria. We analyzed the effects from the studies using a meta-analytic multivariate model and considered the potential moderating effect of multiple variables. Our analysis revealed exercise to decrease the feelings of fatigue by a small effect size (*g* = −0.374; 95% CI [−0.521, −0.227]), increase energy by a small-to-moderate effect size (*g* = 0.415; 95% CI [0.252, 0.578]), and to increase the feeling of vitality by a moderate effect size (*g* = 0.537; 95% CI [0.404, 0.671]). All main results remained robust after several sensitivity analyses using different statistical estimators, and consideration of outlier and influential studies. Moreover, moderator analyses revealed significant effects of exercise intensity and intervention duration on fatigue, exercise intensity, and modality on energy, and participant health, exercise intensity modality, and exercise training location on vitality. We conclude that when groups adopt a moderate intensity exercise training program while participating in a randomized trial, compared to controls, this typically results in small-to-moderate average improvements in feelings of fatigue, energy, and vitality.

## Introduction

Energy and fatigue are complex constructs with emotional, behavioral, and cognitive components that are strongly related to health and quality of life (O’Connor and Puetz, 2005). Energy and fatigue have been conceptualized as static personality traits (Manierre et al., 2020) or dimensions of cognitive effort (Mathews and MacLeod, 2005). In other ways, energy and fatigue have been conceptualized as symptoms of other physiological processes (Lewis and Wessely, 1992), such as sleep (Hardy and Studenski, 2008) and illnesses (De Groot et al., 2003), or as an index of subjective well-being (Ware and Sherbourne, 1992). In this review, fatigue, energy and vitality, a measure that combines feelings of energy and fatigue, are defined as transient mood states that can be affected by behavioral interventions, but are enduring enough to cause downstream physiological (Heuchert and McNair, 2012), affective, cognitive, and behavioral (Forgas, 2020) consequences. Specifically, we have defined the mood of energy as positive feelings regarding the capacity to complete mental or physical activities and the mood of fatigue as negative feelings regarding a reduced capacity to complete mental or physical activities (O’Connor and Puetz, 2005).

There is a much larger body of research investigating prevalence, causes, and treatment approaches for fatigue in chronic health conditions than on energy, although the two are inherently linked. For example, many review articles have been published supporting high prevalence and impact of fatigue in cancer patients (Narayanan and Koshy, 2009), persons with neurological disorders (e.g., multiple sclerosis; Chaudhuri and Behan, 2004), somatic disorders (e.g., chronic fatigue syndrome; Barsky and Borus, 1999), and autoimmune illnesses (e.g., rheumatoid arthritis; Zielinski et al., 2019). Fatigue is also a common complaint in otherwise healthy individuals (Pfaff, 2006) and has even been linked with an increased risk of cardiac events (Kop et al., 1994). Current treatments for reducing fatigue are not overwhelmingly successful and results are highly heterogeneous (Patrick et al., 2003; Pfaff, 2006). Pharmacological treatments, such as Modafinil (e.g., Provigil), Amantadine (e.g., Gocovri), Dextroamphetamine (e.g., ProCentra), and Methylphenidate (e.g., Daytrana), are inconsistently effective, especially with continued, long-term use (De Groot et al., 2003; DeBattista et al., 2003; Asano and Finlayson, 2014; Miller and Soundy, 2017). Given the high prevalence of feelings of significant fatigue, there is a need for non-pharmacological long-term treatment modalities that are accessible, safe, and efficacious.

Physical activity (PA) or structured exercise (i.e., planned PA to improve health-related fitness) may be an effective non-pharmacological treatment option to improve fatigue. Cross-sectional studies support that higher fatigue is related to poorer physical function, lower cardiorespiratory fitness, and lower PA (Stewart et al., 1994; Coakley et al., 1998; Hong and Dimsdale, 2003). Research on the impact of increased PA on feelings of fatigue in healthy sedentary individuals suggests positive benefits, although results are mixed and there may be a floor effect if participants are not experiencing significant fatigue at the start of an exercise training program (Dyer and Crouch, 1987; O’Connor and Puetz, 2005). There is a limited amount of evidence for efficacy of multidimensional treatment modalities for fatigue when PA is combined with drug or cognitive-behavioral therapy (Fillion et al., 2008; Miller and Soundy, 2017). Rehabilitation programs based on increased PA alone have demonstrated a moderate effect on fatigue in patients with multiple sclerosis (Asano and Finlayson, 2014) during and following cancer treatment (Dimeo et al., 1999; Puetz and Herring, 2012; Meneses-Echávez et al., 2015; Hong et al., 2020) as well as small effects on people with Lyme disease (D’Adamo et al., 2015). However, not all studies show clear positive effects and many investigations have been marred by methodological shortcomings (Neill et al., 2006; Braam et al., 2016).

A previous meta-analysis found that acute exercise (i.e., a single bout of exercise for about 20–40 min) consistently increases feelings of energy but feelings of fatigue are reduced primarily after ≥20 min of low-to-moderate intensity exercise that concurrently increased feelings of energy (Loy et al., 2013). This complex finding supports the need for including both feelings of energy and fatigue in exercise research. Our earlier narrative review found that exercise training programs improve feelings of energy and fatigue, but also identified methodological concerns such as no study including a placebo (i.e., attention only) group and control group (O’Connor and Puetz, 2005). Our subsequent meta-analysis analyzed 70 experimental studies of exercise training on energy and fatigue and found a mean standardized post-training improvement of 0.37 (Puetz et al., 2006). However, a significant moderator was the presence of a placebo condition and the majority of studies analyzed involved older adults with chronic medical conditions. Also, energy and fatigue were combined into one single effect which failed to consider subtle differences in the effects of exercise training on energy versus fatigue. Therefore, as the amount of research in this area has increased and the methodological rigor has improved in studies published since 2006, an updated meta-analysis of randomized controlled trials (RCTs) is warranted using meta-analytic multivariate methods that enable the inclusion of a greater number of effects and consequently, more precise outcome estimates.

The purpose of this systematic review and meta-analysis is to systematically review RCTs that have investigated the effects of chronic exercise/exercise training programs on mood states of fatigue, energy, and vitality. While energy and fatigue are defined as separate, but related moods, vitality is a composite construct related to the perception of both energy and fatigue (Stansfeld et al., 1997). Vitality is a very widely assessed construct that measures the frequency of a bipolar energy-fatigue mood state. Vitality is most commonly measured using the SF-36 Health Survey (SF-36). The intensity of current energy and fatigue states are most commonly measured using separate, unipolar items on the Profile of Mood States (POMS). A large body of evidence supports the reliability and validity of all three of these self-report metrics (O’Connor, 2004).

The primary purpose of this analysis is to quantify the magnitude and variability of the effect of chronic exercise, within the context of RCTs, on feelings of fatigue, energy, and vitality. The secondary purpose is to identify sample, methodological, and exercise-based characteristics that explain some of the anticipated heterogeneity in the results (i.e., moderate the effects). Based on previous research, we hypothesize small to moderate negative effects on fatigue and small to moderate positive effects on energy and vitality. Further, we hypothesize significant moderators to be participant fitness and health, exercise intensity, and the type of control/comparison group used.

## Methods

### Literature Search and Management

All items in this protocol correspond with the Preferred Reporting Items for Systematic Review and Meta-Analysis Protocols Statement (PRISMA-P; Moher et al., 2016). Corresponding to the PRISMA guidelines (Shamseer et al., 2015; Moher et al., 2016), our review protocol was registered with the Open Science Framework on 9 March 2021 (Claesen et al., 2021). Registration number and link: 10.17605/OSF.IO/RQ82B.

The literature used in this meta-analysis was obtained before December 2021 from the following electronic databases: PUBMED, PsycINFO, SPORTDiscus, Web of Science, ProQuest Dissertations and Theses, and Google Scholar (500 first hits). The first and the second authors (CW and MM) gathered the literature from the databases using the following keywords: *exercise, training, physical activity, resistance, strength, aerobic, chronic, fatigue, energy, vigor, vitality*. The following search string was used for all databases: {[fatigue AND (energy OR vigor)] OR vitality} AND (chronic OR training) AND (exercise OR physical activity OR aerobic OR resistance).

In addition to the database search, the reference lists of all included studies and relevant review studies found in the search were scanned. Moreover, a backward search using the *Cited by* and *Related articles* tabs in Google Scholar was conducted with all the included studies. Lastly, all the corresponding authors of the included articles were contacted *via* email or in Research Gate^1^ up to three times to request unpublished research or research that was not located with the other search methods.

**FIGURE 1:**
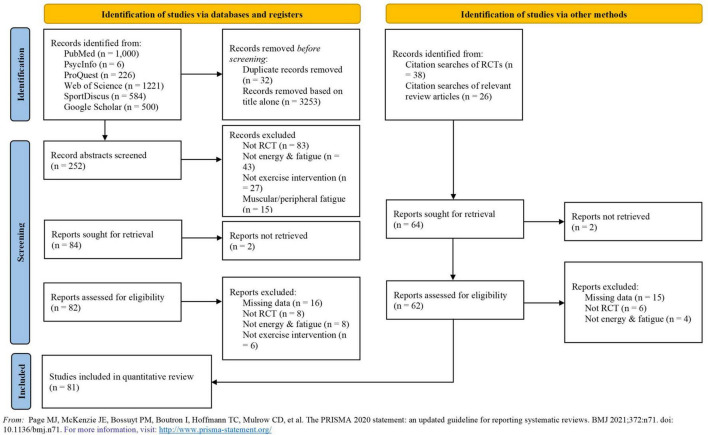
PRISMA flow diagram. Detailed flow of studies examined from the initial search to the final inclusion.

Each article found from the different searches was scanned independently by the first two authors (CW and MM) by applying the inclusion and exclusion criteria to the title/abstract. All duplicate articles were removed. Each study carried forward from this stage was fully read and reviewed independently by the same authors aiming to find the studies to be meta-analyzed. All reasons to exclude studies at this stage were recorded and are displayed in Figure 1.

### Study Selection

The following inclusion criteria were applied: (1) RCTs, (2) studies with at least two data points (pre- and post-measures), (3) samples with a mean age ≥18 years, (4) interventions addressing the effect of chronic exercise or exercise training (i.e., more than one bout of exercise per week across more than 3 weeks), (5) balance training, stretching, and other active controls or non-treatment, usual care and wait-list control conditions, (6) self-report measures of energy and fatigue levels or vitality, (7) articles reported in the English language, (8) peer-reviewed articles, dissertations, book chapters, conference papers, and unpublished research, (9) all research made available prior to December 2021. The exclusion criteria were as follows: (1) cross sectional, case reports, qualitative findings, and non-randomized trials, (2) comparisons to resistance or aerobic exercise, (3) studies using physiological measures or behavioral assessments as the only fatigue, energy, or vitality outcome measures, and (4) studies measuring outcomes during exercise.

### Data Extraction, Moderators, and Risk of Bias

The first two authors independently extracted sample sizes, means, and standard deviations (SD)/standard errors (SE) of the outcome measures from each study. In two studies, the desired effect sizes and their variance were pulled directly from the manuscript. Moreover, in the case of four studies, the statistical information was extracted from study figures using ImageJ (Schneider et al., 2012). In addition, we estimated the mean and SD from one study based on the median and interquartile range values as suggested by the Cochrane handbook (Higgins et al., 2022). Lastly, for two studies, we imputed missing SD from the most similar study. All the described techniques were applied when we did not receive the missing information from the study authors as suggested by the Cochrane handbook (Higgins et al., 2022). Interrater agreement for all extracted data used in effect size calculation was assessed as an unweighted Cohen’s kappa for categorical data and with an intraclass correlation coefficient (ICC) for continuous data. Any dissimilarities were located and resolved before the final calculations were completed.

Besides quantitative information, we extracted *a priori* moderators, including characteristics of the experimental interventions (setting, mode of exercise, frequency, duration, and intensity) and control condition (type), participant characteristics (mean age, gender, adherence to the intervention, physical activity and fitness levels, and health status), details of the applied self-report instruments, and features of the paper (country, publication status, and publishing year). Interrater agreement for all coded moderators analyzed was assessed as an unweighted Cohen’s kappa. Any dissimilarities were located and resolved before the final calculations were completed.

Risk of bias was assessed for each study using the Cochrane Risk of Bias tool (Brock and Carter, 2017; Carter et al., 2019). This tool covers sequence generation, allocation concealment, blinding, incomplete outcome data (i.e., dropouts), and selective outcome reporting. The risk for each domain was rated as high risk, low risk, or unclear. The strength of the overall evidence was also assessed (Sterne et al., 2019). Lastly, the certainty of evidence for all outcomes was assessed using the GRADE approach by evaluating five domains, including risk of bias, inconsistency, indirectness, imprecision, and publication bias (Schünemann et al., 2013).

### Effect Size Calculation

Outcomes were analyzed as standardized mean change differences (Hedges’ *g*) between the exercise and control conditions in R (version 4.0.2; R Core Team, 2018) using the escalc function in the metafor package (Viechtbauer, 2010). To begin, we calculated the standardized mean changes from pre- to post-test for both conditions using the pre-test SD and a bias correction factor (Becker, 1988). As the pre–post-test correlations were not available in the studies, we used an estimate correlation of 0.7 to compute the standardized mean changes, while also testing alternative correlations of 0.5 and 0.9. Next, the standardized mean change difference was computed by subtracting the standardized mean change of the exercise conditions from the corresponding statistic of the control conditions (Morris, 2008). The effect size sampling variances were generated by summing up the sampling variances of both conditions. An increase in feelings of fatigue resulted in a negative effect size, while an increase in feelings of energy and vitality resulted in a positive effect size.

### Statistical Analysis

All meta-analytical procedures were conducted in R (version 4.0.2; R Core Team, 2018) using a maximum likelihood multivariate random effects model with the metafor package (Berkey et al., 1998; Viechtbauer, 2010). The multivariate model was applied as the data had multiple dependencies (multiple outcomes, multiple time points of measurement, and different exercise conditions with the same control condition coming from the same study). To consider the non-independence of the effect sizes, we constructed a variance-covariance matrix (Berkey et al., 1998) and included it in the meta-analytic multivariate model. The necessary correlations between the outcomes (vitality, energy, and fatigue) to compute the variance-covariance matrix were taken from the study by Fink et al. (2010) and the autocorrelation between the different time points at a 1-week interval was estimated to be 0.95, with alternative autocorrelations of 0.9 and 0.97 also tested. As the exact magnitude of dependence of the effects was unknown, robust variance estimator from the clubSandwich package was used to improve the accuracy of the estimates (Pustejovsky and Tipton, 2021).

In the multivariate model, random effects were added for each effect size within each study allowing the effect sizes to correlate and have different variances. The between-study heterogeneity of the effects were examined by parameters of *tau*^2^ and *I*^2^ (Higgins et al., 2003; Jackson et al., 2012). Importantly, as the standard heterogeneity statistic *Q* cannot be applied to multivariate models, a likelihood ratio test examining the effect of *tau*^2^ on all the outcomes was used to gauge significant between study heterogeneity. The between study heterogeneity of the effect sizes was specified if likelihood ratio test (χ^2^) reached a significance level of *p* < 0.05, and the sampling error accounted less than 75% of the observed variance (Freeman et al., 1986; Lipsey and Wilson, 2001).

The moderators were used in a linear regression analysis as univariate variables to explain the possible heterogeneous effects of the outcomes. A modified version of the Egger’s test (Egger et al., 1997) using the SE of the observed outcomes as a predictor in a multivariate model and a visual examination of normal and contour enhanced funnel plots were used to detect publication bias (Peters et al., 2008). The presence of outlier and influential studies and effects were analyzed using Cook’s distances and the distribution of studentized residuals (Viechtbauer and Cheung, 2010).

The sensitivity analyses were computed at several levels. First, alternative pre–post correlations in computing the effect sizes as well as different autocorrelations in computing the variance-covariance matrix were examined. Second, the impact of excluding outlier and influential studies and effects were analyzed. Third, different combinations of the two sensitivity analysis protocols were examined. Sensitivity analysis results are displayed in Table 1.

**TABLE 1:** Sensitivity analyses.

Sensitivity analysis procedure	Energy		Fatigue		Vitality	
	Hedge’s *g*	95% CI	Hedge’s *g*	95% CI	Hedge’s g	95% CI
1. Main (auto correlation *r* = 0.95 and prepost correlation *r* = 0.7)	0.42	[0.25, 0.58]	−0.37	[−0.52, −0.23]	0.54	[0.40, 0.67]
2. Auto *r* = 0.9	0.42	[0.26, 0.58]	−0.39	[−0.53, −0.24]	0.55	[0.42, 0.69]
3. Auto *r* = 0.97	0.41	[0.24, 0.57]	−0.36	[−0.52, −0.21]	0.52	[0.39, 0.65]
4. Prepost *r* = 0.5	0.40	[0.23, 0.56]	−0.31	[−0.48, −0.13]	0.50	[0.37, 0.64]
5. Prepost *r* = 0.9	0.40	[0.22, 0.58]	−0.29	[−0.46, −0.12]	0.51	[0.37, 0.65]
6. 3 + 5	0.39	[0.21, 0.57]	−0.27	[−0.45, −0.10]	0.50	[0.36, 0.63]
7. Influential effects removed	0.44	[0.28, 0.59]	−0.38	[−0.55, −0.22]	0.46	[0.34, 0.58]
8. Influential studies removed	0.40	[0.24, 0.56]	−0.40	[−0.58, −0.23]	0.45	[0.33, 0.56]
9. Outlier effects removed	0.40	[0.24, 0.57]	−0.36	[−0.51, −0.21]	0.49	[0.38, 0.61]
10. Outlier studies removed	0.38	[0.22, 0.53]	−0.32	[−0.44, −0.20]	0.46	[0.35, 0.57]
11. 7 + 8 + 9 + 10	0.40	[0.24, 0.57]	−0.35	[−0.51, −0.18]	0.41	[0.30, 0.53]
12. 6 + 10	0.34	[0.16, 0.52]	−0.31	[−0.43, −0.19]	0.46	[0.34, 0.58]
13. 6 + 11	0.38	[0.18, 0.58]	−0.31	[−0.49, −0.14]	0.42	[0.30, 0.53]

## Results

Altogether, 172 effects (*k*) from 81 studies for energy (*k* = 45), fatigue (*k* = 37), and vitality (*k* = 90) were included. The study selection process from the initial search to the final inclusion is depicted in Figure 1. The total number of participants was 7,050 (69% females). The mean (SD) age of the participants was 49.39 (6.41) years. The exercise intervention lasted between 6 and 48 weeks, with a mean (SD) duration of 13.57 (9.98) weeks. Mean (SD) time per session was 47.82 (22.95) min and mean (SD) number of sessions per week was 3.06 (1.10). Most of the analyzed studies employed a non-active control group, including usual care (*k* = 53) or wait list control (*k* = 117), while some studies employed an active control condition [e.g., stretching exercise (*k* = 19)]. Seventeen studies (20.1%) included were also included in a previous meta-analysis (Puetz et al., 2006). The full descriptive information of the included studies is shown in Supplementary Table 1.

**FIGURE 2:**
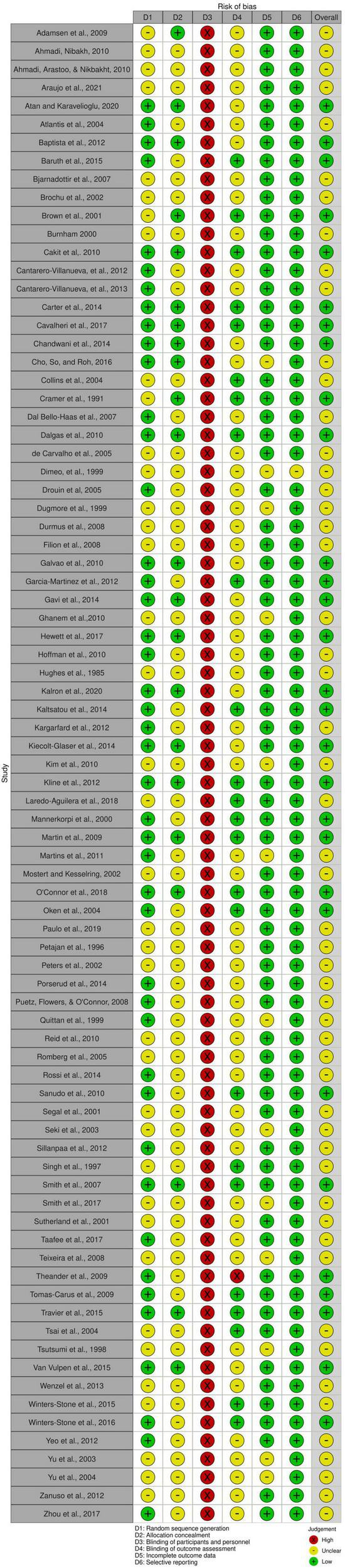
Risk of bias of each included study. The risk of bias of each criterion measured by the Cochrane Risk of Bias tool and the total risk of bias for each individual study. Graphic by the RoB2 tool.

**FIGURE 3:**
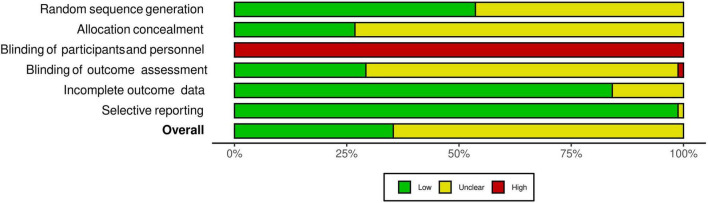
Summary of risk of bias. Summary of risk of bias based on each criterion measured by the Cochrane Risk of Bias tool. Graphic by the RoB2 tool.

The interrater agreement statistics support strong agreement between authors. Initially the absolute agreement between the two first authors for all extracted continuous data using the two-way mixed effect model and “single rater” unit for ICC was 0.98 (95% CI = 0.98, 0.98), *p* < 0.001. The initial interrater reliability for moderator coding *via* unweighted Cohen’s kappa was 0.83 (95% CI = 0.81, 84), *z* = 90.6, *p* < 0.001 with the rough percent agreement being 86.6%. Lastly, in the beginning, the interrater agreement applying the Risk of Bias tool as an unweighted Cohen’s kappa was 0.51 (95% CI = 0.44 to 0.59), *z* = 12, *p* < 0.001 with a rough percentage agreement of 75.2%. All differences in coding were discussed and resolved until full agreement was achieved. As shown in Figure 2, there were no studies that had a high risk of bias. Most studies were coded as having moderate risk due to missing information and a lack of transparency in the manuscripts. As shown in Figure 3, all studies were ranked as having high risk of bias based on blinding of participants. However, it is almost impossible to blind individuals to an exercise treatment if they are in an exercise program (Smart et al., 2015), so this specific criterion was not included in the study’s total risk of bias score. Regardless, the Cochrane Risk of Bias tool is ubiquitous in systematic reviews and meta-analyses and was therefore used here.

### Effects of Exercise on Feelings of Energy, Fatigue, and Vitality

For fatigue, 84% of the outcome estimates were negative, ranging from −1.53 to 0.371. The multivariate model indicated that the standardized mean change difference between the exercise and control conditions was −0.374 (95% CI [−0.521, −0.227], *t* = −5.302, *p* < 0.001). The standardized mean change difference differed significantly from zero but was heterogeneous [χ^2^(1) = 35.09, *p* < 0.001, *tau*^2^ = 0.146, *I*^2^ = 62.78%].

For energy, 89% of the outcome estimates were positive, ranging from −0.247 to 2.047. The multivariate model indicated that the standardized mean change difference between exercise and the control conditions on energy was 0.415 (95% CI [0.252, 0.578], *t* = 5.263, *p* < 0.001). The standardized mean change difference differed significantly from zero but was heterogeneous [χ^2^(1) = 24.23, *p* < 0.001, *tau*^2^ = 0.178, *I*^2^ = 71.05%].

For vitality, 83% of the outcome estimates were positive, ranging from −0.722 to 3.271. The multivariate model indicated that the standardized mean change difference between exercise and the control conditions on vitality was 0.537 (95% CI [0.404, 0.671], *t* = 8.036, *p* < 0.001). The standardized mean change difference differed significantly from zero but was heterogeneous [χ2(1) = 105.49, *p* < 0.001, *tau*^2^ = 0.259, *I*^2^ = 86.89%].

**FIGURE 4:**
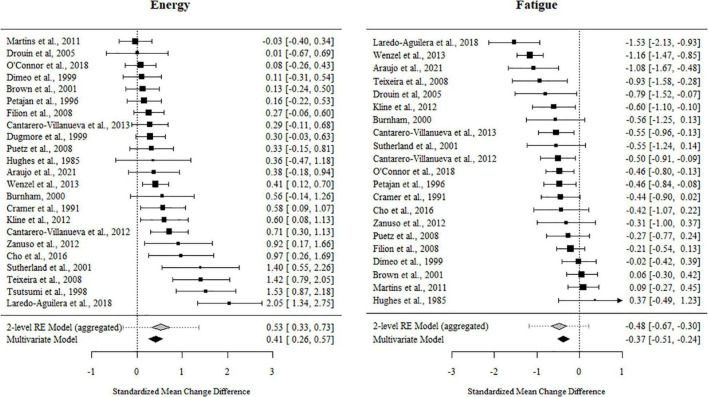
Forest plots for energy and fatigue. Aggregated study effects displayed with a mean estimate coming from a meta-analytic model and a two-level random effects model for comparison reasons.

**FIGURE 5:**
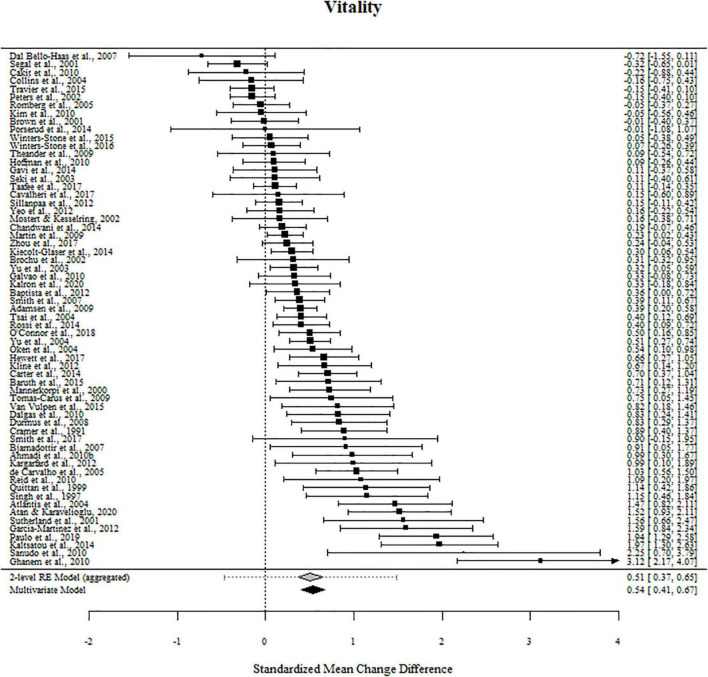
Forest plots for vitality. Aggregated study effects displayed with a mean estimate coming from a meta-analytic model and a two-level random effects model for comparison reasons.

The effect sizes aggregated at the study level (one effect per study displayed per outcome) and their CIs as well as the standardized mean change difference according to a meta-analytic multivariate model and a two-level random effects models are displayed in Figures 4, 5. Influential studies and outliers can be found in Supplementary Figure 1. The aggregated data set and R-code used for analysis can be found on the OSF website. Additional information can be shared upon request.

### Moderator Analyses

As shown in Tables 2, 3, significant moderators of exercise training on fatigue included participant sex, exercise intensity, duration of intervention in weeks, and total time of exercise in minutes. The sex-based differences are likely based on the comparison of one effect that found small increases in fatigue in a small sample of sedentary men following an aerobic exercise intervention (*n* = 14), compared to 36 other effects from studies in which female participants were included that support decreases in fatigue following exercise interventions. The benefits of exercise on fatigue were significantly greater in moderate intensity exercise (*g* = −0.394) interventions than in light intensity exercise (*g* = −0.013) interventions. The effects of exercise on fatigue were significantly related to time in exercise, such that fatigue improved as the duration of exercise training and the exercise session total time increased. Both continuous moderators suggest that as an intervention duration and the total exercise time increases, the benefits of such an intervention on fatigue increase accordingly.

**TABLE 2:** Univariate results for significant fatigue categorical moderator variables.

Effect moderator	Number of effects	Hedge’s *g*	95% CI	*p*-Value
**Participant characteristics**				
**Sex**				
Male only	1	0.407	[−0.185, 0.990]^a^	
Female only	9	−0.392	[−0.561, −0.223]^b^	0.023
Both	27	−0.453	[−0.748, −0.159]^b^	0.045
**Exercise characteristics**				
**Intensity**				
Light	6	−0.013	[−0.132, 0.106]^a^	
Moderate	29	−0.394	[−0.548, −0.240]^b^	<0.001
High	0			

*^a,b^Moderator levels with a non-common superscript differ significantly. p-Value reported is based on a meta-regression analysis.*

**TABLE 3:** Univariate results for significant fatigue continuous moderator variables.

Effect moderator	β	95% CI	*t*-Value	*p*-Value
Duration (weeks)	−0.032	[−0.054, −0.009]	−3.340	0.015
Total time (min)	−0.0001	[−0.002, −0.001]	−5.157	0.004

*p-Value reported is based on a meta-regression analysis. Superscripts and p-values are reported only for significantly different moderators.*

As shown in Table 4, significant moderators of exercise training on energy included exercise intensity and exercise modality. The effect of moderate intensity exercise (*g* = 0.418) was significantly greater on energy than light intensity exercise (*g* = −0.047). It is possible that high intensity exercise was also significantly more effective on energy than light intensity exercise, although energy was only measured in one study that employed high intensity exercise. Exercise interventions that contained components of resistance training, either alone (*g* = 0.569) or combined with aerobic exercise (*g* = 0.636), were significantly more beneficial on energy than aerobic exercise alone (*g* = 0.210).

**TABLE 4:** Univariate results for significant energy categorical moderator variables.

Effect moderator	Number of effects	Hedge’s *g*	95% CI	*p*-Value
**Exercise characteristics**				
**Intensity**				
Light	6	−0.047	[−0.199, 0.104]^a^	
Moderate	36	0.418	[0.231, 0.605]^b^	<0.001
High	1	0.430	[0.231, 0.569]^b^	<0.001
**Modality**				
Aerobic	28	0.210	[0.067, 0.353]^a^	
Resistance	5	0.569	[−0.100, 1.241]^b^	0.021
Combined	11	0.636	[0.300, 0.972]^b^	0.018

*^a,b^Moderator levels with a non-common superscript differ significantly. p-Value reported is based on a meta-regression analysis. Superscripts and p-values are reported only for significantly different moderators.*

As shown in Table 5, significant moderators of exercise training on vitality included participant health, exercise intensity, exercise modality, and exercise location. The effects of exercise training on vitality were significantly greater in persons with neurological disorders (*g* = 0.614) than in persons with cancer (*g* = 0.295). The average effect of exercise training on vitality was moderate-sized in the moderate intensity interventions (*g* = 0.503), significantly greater in light intensity (*g* = 0.608) interventions, and significantly highest in high intensity (*g* = 0.803) interventions. However, the intensity-based differences may be based in part on the inequity of effects, since the majority of studies used a moderate intensity intervention (*k* = 62), compared to very few that employed a light (*k* = 3) or high intensity intervention (*k* = 6). Results suggest that combining modes may be more effective than an aerobic intervention alone, although differences were not statistically different from resistance training alone. Finally, the effects of exercise on vitality were significantly less in studies that prescribed both unsupervised home-based exercise and supervised facility-based exercise (*g* = 0.276) compared to supervised exercise in a facility alone (*g* = 0.589).

**TABLE 5:** Univariate results for significant vitality categorical moderator variables.

Effect moderator	Number of effects	Hedge’s *g*	95% CI	*p*-Value
**Participant characteristics**				
**Health status**				
Healthy	13	0.657	[0.245, 1.068]	
Cancer	24	0.295	[0.084, 0.507]^a^	
Heart disease	12	0.533	[0.043, 1.024]	
Neurological disorder	28	0.614	[0.368, 0.860]^b^	0.042
Other	13	0.577	[0.213, 0.941]	
**Exercise characteristics**				
**Intensity**				
Light	3	0.608	[0.440, 0.775]^a^	
Moderate	62	0.503	[0.352, 0.654]^b^	0.009
High	6	0.803	[0.584, 1.022]^c^	<0.001
**Modality**				
Aerobic	32	0.393	[0.259, 0.526]^a^	
Resistance	19	0.522	[0.280, 0.763]	
Combined	29	0.644	[0.428, 0.860]^b^	0.009
Yoga	8	0.562	[0.334, 0.790]	
Other	2	0.849	[−3.752, 5.45]	
**Location**				
Home	12	0.569	[0.266, 0.871]	
Facility	61	0.589	[0.435, 0.743]^a^	
Both	17	0.276	[0.040, 0.511]^b^	0.045

*^a,b,c^Moderator levels with a non-common superscript differ significantly. p-Value reported is based on a meta-regression analysis using the ^a^moderator level as the reference point.*

### Publication Bias

Publication bias (i.e., publication of studies with results of certain type and direction) was examined by visual examination of normal and contour-enhanced funnel plots using a modified Egger’s test (Egger et al., 1997). For the funnel plots, an aggregated effect from each study was plotted so that only one effect size per study per outcome is displayed.

**FIGURE 6:**
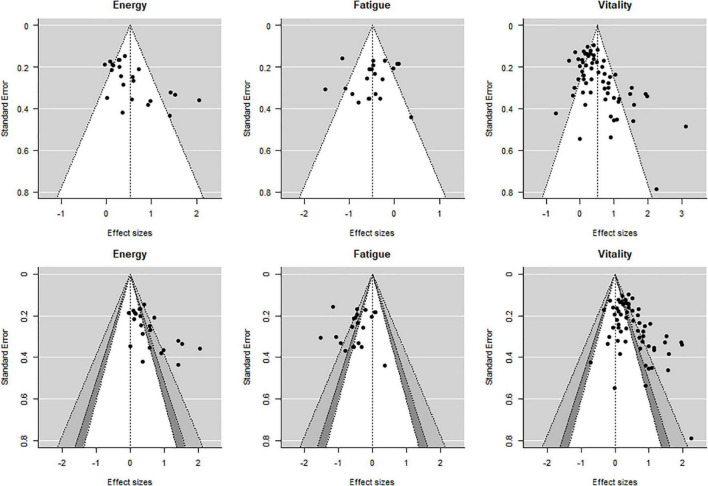
Normal and contour enhanced funnel plots. Aggregated effects (one per study per outcome). Shading for the bottom row: white: 0.10 < *p* < 1.00, dark gray: 0.05 < *p* < 0.10, medium gray: 0.01 < *p* < 0.05, and lightest gray: 0.00 < *p* < 0.01.

The modified Egger’s test for the regression intercept was found to be significant for energy (*p* = 0.10) and vitality (*p* < 0.001) but not for fatigue (*p* = 0.092) suggesting publication bias for energy and vitality. Similarly, asymmetry of the different funnel plots for energy and vitality suggests publication bias for these two outcomes (Figure 6).

As shown in Table 6, the certainty of evidence for all studies was moderate based on the GRADE approach. Given the large number of studies and effects, some heterogeneity and variance is expected. Given the criteria using the GRADE approach, the observed heterogeneity is a minor influence on the certainty of the evidence for fatigue, energy, and vitality.

**TABLE 6:** Summary of findings table.

Certainty assessment							No. of patients		Effect		Certainty	Importance
No. of studies	Study design	Risk of bias	Inconsistency	Indirectness	Imprecision	Other considerations	Exercise training	Non-exercise	Relative (95% CI)	Absolute (95% CI)		
**Fatigue (assessed with: questionnaire)**												
21	Randomized trials	Not serious	Serious^a^	Not serious	Not serious	None	550	566	-	SMCD 0.374 SD lower (0.521 lower to 0.227 lower)	⊕⊕⊕○ Moderate	IMPORTANT
**Energy (assessed with: questionnaire)**												
21	Randomized trials	Not serious	Serious*^a^*	Not serious	Not serious	None	636	590	-	SMCD 0.415 SD higher (0.252 higher to 0.422 higher)	⊕⊕⊕○ Moderate	IMPORTANT
**Vitality (assessed with: questionnaire)**												
21	Randomized trials	Not serious	Serious*^a^*	Not serious	Not serious	None	2,677	2,031	-	SMCD 0.537 SD higher (0.404 higher to 0.671 higher)	⊕⊕⊕○ Moderate	IMPORTANT

*^a^Large and significant heterogeneity, all effects are not in the same direction.*

## Discussion

### Magnitude of the Effect

Chronic exercise, or exercise training interventions, were associated with significant improvements in fatigue, energy, and vitality. The magnitude of effects (*g* = −0.374, 0.415, and 0.537, respectively) is greater than that previously reported on combined energy and fatigue in 2006 (Δ = 0.37; Puetz et al., 2006). Such a magnitude of effect is greater than well-established effects of chronic exercise on other aspects of mental health such as cognitive function in healthy older adults (*g* = 0.21; Chen et al., 2020), self-report anxiety in both mid-aged adults (Δ = 0.31; Gordon et al., 2017) and older adults with medical issues (Δ = 0.29; Herring et al., 2010), and chronic pain in sufferers of chronic lower back pain (SMD = −0.32; Searle et al., 2015). These effects also support previous assertions that exercise is more effective at improving fatigue, energy, and vitality than cognitive-behavioral therapy or pharmacologic treatments (Puetz et al., 2006). Despite the aforementioned greater research focus on fatigue in studies of people with chronic health conditions, the effects found here suggest that energy and vitality are just as important to measure, especially in evaluating potential treatment approaches. In addition, the varying effect sizes in the present analysis support that fatigue, energy, and vitality are distinct yet related constructs as has been suggested by other types of evidence (Boolani et al., 2019) and future studies should consider measuring fatigue, energy, and vitality when seeking a full evaluation of treatment interventions and/or exercise training protocol (i.e., resistance, aerobic, or a combination). For example, our results suggest that exercise training has a larger effect on the perceptions about the *frequency* of energy and fatigue feelings combined rather than on the *intensity* of energy and fatigue separately. This interpretation comes from the larger effect size for vitality, which queries prior month frequency, than either energy or fatigue alone, which measured current feelings. The dominant measures of energy and fatigue alone ask respondents about the *intensity* of feelings. The intensity of energy and fatigue feelings may be less memorable than recollections about the frequency of feelings of energy and fatigue as has been found for the accuracy of memory for other symptoms (Thomas et al., 2011).

### Primary Moderators of the Effect

Characteristics of the exercise intervention independently moderated the effects of exercise on fatigue, energy, and vitality. While the majority of studies employed a supervised exercise program in a facility, some prescribed an at-home intervention or a combined supervised and home intervention. For vitality, a supervised, facility-based intervention was significantly more effective compared to studies that combined facility and at-home prescription. One possible explanation for this finding is that studies that used both facility and at-home prescription may have been longer and therefore resulted in lower adherence which attenuated the effect.

For both energy and vitality, greater improvements occurred in those studies that combined resistance exercise with aerobic exercise compared to aerobic exercise alone. This finding is consistent with the Physical Activity Guidelines for Americans which recommends that both aerobic and muscle strengthening activities be performed regularly by adults to support health, including mental health outcomes (Piercy et al., 2018). One plausible explanation for this finding is that the total exercise dose was greater when the two modalities were combined compared to aerobic activities alone. One relevant review compared aerobic training to strength training studies examining the effects on perceived fatigue in persons with multiple sclerosis and found no difference in effect size (Taul-Madsen et al., 2021). No RCT to date has directly compared an aerobic exercise training to a resistance exercise training program on energy, fatigue, or vitality. Direct comparisons, however, are likely to be imperfect because of fundamental differences in the two modes. For example, continuous vs. non-continuous muscle actions in aerobic vs. resistance modes, respectively means that if the two modes are matched on total session duration then the amount of work completed is greater in the aerobic mode unless the two groups differ greatly on exercise intensity. Alternatively, if the session duration is held constant and two modes are matched on relative exercise intensity then the total work differs because of the rest that occurs with resistance exercise. Moreover, if matched on total work then the relative intensity and/or session durations differ (Herring et al., 2011).

For fatigue, energy, and vitality, exercise intensity is a significant moderator, but the comparisons are difficult to interpret because of the relatively small number of effects involving light and high intensity exercise. The dozens of effects for moderate intensity yielded the consistent finding that moderate intensity exercise training induces moderate sized improvements in feelings of fatigue, energy, and vitality. A much smaller body of evidence suggests that low intensity exercise does not improve feelings of energy and fatigue, but these estimates are imprecise because they are based only six effects for each outcome. It is possible that longer session durations of low intensity exercise or longer program durations may be needed to produce significant improvements in fatigue, energy, and vitality. Additional research on this topic would be welcome, in part because many people, including those with disabilities or medical illnesses, can safely perform only low intensity exercise and others prefer low intensity exercise. High intensity exercise appeared to improve vitality to a greater extent than moderate intensity exercise, though this maybe unreliable because it was based on only six effects. Given the increased research and clinical interest in time efficient, high intensity exercise, there is a need to fill the research gap concerning the potential influence of regular, brief high intensity bouts of exercise on feelings of fatigue, energy, and vitality (Martland et al., 2020).

For fatigue only, there was a significant moderating effect of the intervention duration in weeks, such that interventions of longer duration showed greater benefits on fatigue. Relatedly, a similar relationship was seen for total number of minutes of exercise in the intervention. Exercise session duration and number of bouts per week, however, were not significant moderators. Thus, the intervention duration appears to be an important consideration when prescribing exercise to mitigate fatigue in people with chronic health conditions. The duration of most RCTs is less than 1 year, hence the current finding must be considered in the context that most of the trials have been relatively short in duration. Exercise adherence is reduced as trials extend to 6-months and beyond, so prescribing longer duration interventions to reduce fatigue is predicated on using theory-based interventions that will maintain adequate adherence (Voth et al., 2016; Room et al., 2017).

Participant health was a significant moderator for vitality only. The effect of exercise was significantly more impactful on vitality in persons with neurological disorders compared to those with cancer. In this analysis, the majority of studies on persons with neurological disorders were those with multiple sclerosis (11/22 studies and 23/38 effects). The second most studied neurological group was persons with fibromyalgia (7/22 studies and 10/38 effects). The smaller effect of exercise in samples with cancer may stem from the sample heterogeneity in those studies. Not only were there several differences in specific cancer type (e.g., breast, prostate, and bladder) but also in stage of treatment (e.g., in remission, in treatment, and post-treatment). Due to the complexity of cancer as a diagnosis, several review articles and meta-analyses have been published on exercise-based treatments for fatigue in persons with cancer (Velthuis et al., 2010; Cramp and Byron-Daniel, 2012). It may be worthwhile to conduct a similar meta-analysis just on fatigue, energy, and vitality from exercise interventions in cancer samples only. Then, cancer-specific characteristics could be analyzed as potential moderators of these three outcomes.

### Secondary Moderators of the Effect

Several moderators chosen *a priori* were not significant moderators of fatigue, energy, or vitality. Samples with chronic health conditions were categorized as either being in treatment, having finished treatment, or not needing treatment. Exercise was equally beneficial regardless of this treatment status. Additionally, the benefits were similar if exercise was prescribed as the lone treatment or within a multimodal treatment program. Therefore, exercise should be incorporated into more inpatient and mid-treatment programs. It appears to be never too early or too late to exercise for mood-related benefits and exercise does not have to be the sole focus of the intervention. In addition, type of control condition (i.e., no treatment or wait list, usual care, and active control group) was not a significant moderator. This is an important outcome as there is constant disagreement in the field of exercise psychology as to what the most appropriate control or comparison condition is for adequately interpreting the results of an exercise intervention.

There were other variables that we expected would be significant moderators but were not consistently reported, and therefore could not be analyzed. Pre-intervention aerobic fitness and leisure time physical activity were sporadically reported. For individuals who are regularly active, it is unlikely that a short moderate intensity exercise intervention would result in significant changes in energy and fatigue. Additionally, adherence was inconsistently reported but is crucial to ensure that the entire prescribed dose of exercise was in fact completed. Very few studies that measured cardiorespiratory fitness using an incremental exercise test did so at baseline and follow-up to evaluate changes in fitness. For aerobic exercise interventions, that manipulation check would support that the exercise prescription was delivered correctly and potential relationships between fitness changes and changes in fatigue, energy, and vitality, could be separated.

### Update From 2006

The previous 2006 meta-analysis concluded with four recommendations for future researchers (Puetz et al., 2006). This meta-analysis provides data regarding how well those shortcomings were addressed.

The first recommendation was to identify specific samples that might benefit most from exercise-based interventions for energy and fatigue. Based on this meta-analysis, persons with multiple sclerosis (Harrison et al., 2021; Torres-Costoso et al., 2021) and fibromyalgia (Estévez-López et al., 2021) may be appropriate groups to target with exercise interventions. In their manuscript, Puetz et al. (2006) suggest that researchers should not just take into account the general health of participants (i.e., the absence of a chronic health condition), but also current psychological status as well. One next step is to categorize the articles analyzed in this meta-analysis by health condition and look more deeply into other symptoms and factors, such as duration of condition, current treatment status, and current symptom severity. Another suggestion to better understand the health of the samples that will benefit most is to recruit individuals with specific combinations of symptoms and compare them, such as persons with multiple sclerosis and a co-morbid depression or anxiety disorder compared to those with multiple sclerosis not suffering from depression or anxiety.

The third recommendation was to explore exercise as one component of a multimodal treatment intervention or exercise combined with other efficacious treatments. In this meta-analysis, we investigated treatment modality as a moderator, such that we compared studies in which exercise was employed as a single treatment modality to studies in which exercise was employed within a larger multimodal program. This characteristic was not a significant moderator. Therefore, moderate intensity exercise training improves fatigue, energy, and vitality regardless of whether it is administered alone or with other treatments.

The fourth recommendation was that more systematic research was required to rule out experimental artifacts, such as the placebo effect, as an explanation for benefits of exercise on energy and fatigue. In this meta-analysis of RCTs, type of control condition (i.e., active/placebo, wait list control, and usual care) was a non-significant moderator. The other important recommendation made by those authors was for greater transparency and description of the placebo condition to determine the optimal placebo control for chronic exercise interventions. Descriptions of the active control or usual care conditions often were not detailed enough for others to replicate. Within those studies that employed an active control condition, 7/12 used stretching with or without relaxation. Within the studies that used an active control condition, there were not enough of each type (e.g., muscle relaxation, placebo vitamins, stretching, and relaxation) to usefully make comparisons among the groups.

The second recommendation was the only one that we feel has not been adequately addressed and that we echo as a recommendation for future researchers. Puetz et al. (2006) recommended that researchers manipulate dimensions of the exercise stimulus (e.g., mode and intensity) to learn the relative importance of each characteristic. Only then can we determine the optimal exercise prescription for fatigue, energy, and vitality. The results of this meta-analysis suggest that modality and intensity are important factors and should be experimentally manipulated in future research.

### Future Directions

There is clearly a need for more studies that manipulate and evaluate exercise intensity as a moderating variable of energy and fatigue. As the popularity of high intensity exercise (e.g., high-intensity interval training; HIIT) has grown exponentially in the past two decades, an emerging body of evidence supports different intensity-dependent physiological effects of exercise that may explain such a moderating effect. Compared to moderate-intensity exercise programs, high intensity training results in greater improvements in aerobic capacity (Rognmo et al., 2004) and heart-rate variability (Alansare et al., 2018). Such differences may stem from differential hormonal responses to moderate- versus high-intensity exercise (Peake et al., 2014) or increased oxidative stress and subsequent cardiorespiratory changes induced by higher intensity exercise (Ye et al., 2019). It is important to dedicate more future resources to confirm the aforementioned results suggesting greater vitality improvements after high intensity exercise training programs because people may be deterred by the immediate exacerbation of fatigue. However, if high intensity exercise shows greater improvement on energy and fatigue chronically, as well as physiological benefits and efficiency, it may become more widely utilized.

In addition, more research is warranted to better understand differences in energy and fatigue between athletes and non-athletes. A likely moderating variable between non-athletes exercising for health and athletes training for competition is the much longer session durations and weekly frequency that athletes pursue which leaves less time for recovery. The current results regarding exercise intensity as a moderating variable for vitality may not translate directly to athletes as it is well established that high intensity exercise worsens feelings of fatigue and energy in samples of athletes (Raglin et al., 1991). Changes in mood during training in athletes are less straightforward as they are influenced by the current phase of training (i.e., pre-season and competition; Malone et al., 2017), length of the season (Lac and Maso, 2004), and periodization of training (i.e., different periods of intensity throughout training to avoid overtraining; Vetter and Symonds, 2010; Hamlin et al., 2019). Based on this systematic review and meta-analysis, the focus on research involving exercise, energy, and fatigue has focused heavily on individuals with chronic illnesses in which greater fatigue may be a symptom. However, prolonged periods of heavy exercise training performed by athletes can produce large reductions in feelings of energy and large increases in feelings of fatigue (Morgan et al., 1987; Meeusen et al., 2013), similar to chronic fatigue syndrome. Therefore, a greater focus should be paid to understanding energy and fatigue shifts throughout an athlete’s year to best avoid worsened fatigue.

The experiments analyzed here were not focused on testing neurobiological mechanisms underlying the influence of chronic exercise on feelings of fatigue, energy, and vitality. Nonetheless, it is worth noting that these effects plausibly result from multiple, concomitant physiological adaptations in the brain as suggested primarily from experiments with rodents. Fatigue-reducing exercise has been associated with reductions in pro-inflammatory cytokines (e.g., interleukin 6) and other markers of inflammation presumably reflecting changes in brain neurons or microglia (LaVoy et al., 2016; Chaves-Filho et al., 2019). Exercise also increases myokine levels in the periphery and some of these proteins, such as myokine cathepsin B, can cross the blood–brain interface (BBI) and help to increase brain-derived neurotrophic factors that plausibly could improve feelings of energy and fatigue (Pedersen, 2019). Glucose is the primary source of energy for the brain and inadequate glucose or insulin resistance are thought to contribute to mood dysregulation (Nguyen et al., 2018). In mice, exercise enhances the transport of insulin across the BBI which potentially could improve feelings of energy and fatigue if these results translate to humans (Brown et al., 2022). Exercise increases brain norepinephrine and dopamine levels and alters the density of receptors for these neurotransmitters in areas of the brain thought to be involved in feelings of energy and fatigue such as the prefrontal cortex, striatum, and nucleus accumbens (Meeusen and De Meirleir, 1995; Stahl, 2002). These biological changes, while strong candidate contributors to improvements in feelings of energy and fatigue, are part of larger, complex set of interacting neural networks that are only just beginning to be understood (Qi et al., 2019; Won et al., 2021). More research evaluating potential mechanisms by which exercise influence energy and fatigue in humans is warranted given that much of the evidence to date comes from rodent studies.

## Conclusion

Methodologically rigorous RCTs demonstrate that, when compared to control conditions, moderate intensity exercise interventions of at least 6 weeks are on average beneficial for fatigue, energy, and vitality in healthy individuals and in those with chronic health conditions. Many unanswered questions remain, and future exercise-based studies should employ measures of fatigue, energy, and vitality, in order to obtain a thorough assessment of these important mood states. The outcome measures most commonly used (i.e., POMS and SF-36) are short and easy to administer. Even if mood states are not the primary outcomes of interest, they are relatively low-burden exploratory measures that are important patient reported outcomes. Moreover, potential placebo responses are arguably reduced when fatigue, energy, and vitality are not the primary outcomes. With continued experimentation and analysis, we can better define samples and individuals who are most likely to benefit from exercise training and learn the optimal exercise prescription that will result in the greatest fatigue, energy, and vitality benefits for different populations. Low energy and fatigue are co-morbid symptoms of many health problems for which exercise is a potentially beneficial treatment. Therefore, there is a large body of research being conducted that could fruitfully tap into fatigue, energy, and vitality mood state data and enhance our understanding of this research area.

## Data Availability Statement

The original contributions presented in the study are included in the article/Supplementary Material, further inquiries can be directed to the corresponding author.

## Author Contributions

CW, MM, and PO’C: conceptualization and writing—review and editing. CW and MM: methodology, data curation, and writing—original draft preparation. MM: statistical analysis. All authors have read and agreed to the published version of the manuscript.

## Conflict of Interest

The authors declare that the research was conducted in the absence of any commercial or financial relationships that could be construed as a potential conflict of interest.

## Publisher’s Note

All claims expressed in this article are solely those of the authors and do not necessarily represent those of their affiliated organizations, or those of the publisher, the editors and the reviewers. Any product that may be evaluated in this article, or claim that may be made by its manufacturer, is not guaranteed or endorsed by the publisher.

## Footnotes

1
www.researchgate.com

## Supplementary Material

Supplementary Figure 1 Influential and outlier effects and studies. Cook’s distances and studentized residuals for effects and studies. For Cook’s distances, values exceeding the median plus six times the interquartile range are considered influential. For studentized residuals (effects), cut-off *z*-value is set at 3.44. For studentized residuals for studies, three Chi-squared critical values are displayed [black line, studies with one effect (*z* = 6.63); green line, studies with two effects (*z* = 9.21); blue line, studies with three effects (*z* = 13.28)]. Influential effects: Cramer et al. (1991) – vitality, Brown et al. (2001) – vitality, Sutherland et al. (2001) – fatigue and vitality, Teixeira et al., (2008) – energy, Ghanem et al. (2010) – vitality, Wenzel et al. (2013) – energy and fatigue, Laredo-Aguilera et al. (2018) – energy, O’Connor et al. (2018) – energy and vitality, and Araújo et al. (2021) – fatigue. Influential studies: Cramer et al. (1991), Sutherland et al. (2001), Ghanem et al. (2010), Laredo-Aguilera et al. (2018), and O’Connor et al. (2018). Outlier effects: Ghanem et al. (2010) – vitality and Paulo et al. (2019) – vitality. Outlier studies: Puetz et al. (2008), Ghanem et al. (2010), Wenzel et al. (2013), Laredo-Aguilera et al. (2018), and Paulo et al. (2019).
