# Supplementary material for: The Effect of Chronic Exercise on Energy and Fatigue States: A Systematic Review and Meta-Analysis of Randomized Trials
Source: Front Psychol. 2022 Jun 3;13:907637. doi: 10.3389/fpsyg.2022.907637 (PMC9206544; doi:10.3389/fpsyg.2022.907637)
Supplement: Supplementary file 1 [file Table_1.DOCX]

Supplementary Table 1. Selected characteristics of individual studies

| Authors | #Effects | Sample | Measure | Control | Mode | Intensity | Frequency (min x session x week) |
| --- | --- | --- | --- | --- | --- | --- | --- |
| (Adamsen *et al.*, 2009) | 1 | Cancer | SF-36 | Usual Care | Aerobic + Resistance | High | 90 x 3 x 6 |
| (Ahmadi *et al.*, 2010; Ahmadi, Arastoo and Nikbakht, 2010)** | 2 | Neuro | Other | NT/WL | Aerobic or Yoga | Moderate | 30-65 x 3 x 8 |
| (Araújo *et al.*, 2021) | 2 | Other | POMS | NT/WL | Aerobic + Resistance | Moderate | 40 x 3 x 24 |
| (Atan and Karavelioğlu, 2020) | 2 | Neuro | SF-36 | Usual Care | Aerobic + Resistance | Moderate/High | 35 x 5 x 6 |
| (Atlantis *et al.*, 2004)* | 1 | Healthy | SF-36 | NT/WL | Aerobic + Resistance | High | 20 x 5 x 24 |
| (Baptista *et al.*, 2012) | 2 | Neuro | SF-36 | NT/WL | Other | NR | 60 x 2 x 16 |
| (Baruth *et al.*, 2015) | 1 | Cancer | SF-36 | Usual Care | Aerobic | Moderate | 30 x 4 x 12 |
| (Bjarnadottir *et al.*, 2007) | 1 | Neuro | SF-36 | NT/WL | Aerobic + Resistance | Moderate | 60 x 3 x 5 |
| (Brochu *et al.*, 2002) | 1 | Heart | SF-36 | Active | Resistance | Moderate | 35 x 3 x 24 |
| (Brown *et al.*, 2001)* | 3 | Other | POMS/Other | Active | Aerobic | Moderate | 20 x 5 x 8 |
| (Burnham, 2000) | 4 | Cancer | Other | NT/WL | Aerobic | Moderate | 23 x 3 x 10 |
| (Çakit *et al.*, 2010) | 2 | Neuro | SF-36 | NT/WL | Resistance | Light/High | 90 x 2 x 8 |
| (Cantarero-Villanueva *et al.*, 2012) | 4 | Cancer | POMS | Usual Care | Aerobic + Resistance | Moderate | 55 x 3 x 8 |
| (Cantarero-Villanueva *et al.*, 2013) | 4 | Cancer | POMS | Usual Care | Aerobic | Moderate | 40 x 3 x 8 |
| (Carter *et al.*, 2014) | 2 | Neuro | Other | Usual Care | Aerobic + Resistance | Moderate | 17.5 x 3 x 12 |
| (Cavalheri *et al.*, 2017) | 1 | Cancer | SF-36 | Usual Care | Aerobic + Resistance | Moderate | 60 x 3 x 8 |
| (Chandwani *et al.*, 2014) | 8 | Cancer | SF-36 | Active/NT/WL | Yoga | NR | 60 x 3 x 6 |
| (Cho, So and Roh, 2016) | 2 | Other | POMS | NT/WL | Aerobic | Moderate | 40 x 3 x 8 |
| (Collins *et al.*, 2004) | 1 | Heart | SF-36 | NT/WL | Aerobic | Moderate | 60 x 3 x 24 |
| (Cramer, Nieman and Lee, 1991)* | 6 | Healthy | POMS/Other | NT/WL | Aerobic | Moderate | 45 x 5 x 15 |
| (Dal Bello-Haas *et al.*, 2007) | 1 | Neuro | SF-36 | Usual Care | Resistance | Moderate | NR |
| (Dalgas *et al.*, 2010) | 1 | Neuro | SF-36 | NT/WL | Resistance | Moderate | 60 x 2 x 12 |
| (De Carvalho *et al.*, 2005) | 1 | Neuro | SF-36 | NT/WL | Aerobic | Moderate | 60 x 3 x 12 |
| (Dimeo *et al.*, 1999)* | 2 | Cancer | POMS | Usual Care | Aerobic | Moderate | 30 x 7 x NR |
| (Dugmore *et al.*, 1999) | 6 | Heart | POMS | NT/WL | Aerobic + Resistance | Moderate | NR x 3 x 48 |
| (Durmus *et al.*, 2009) | 1 | Other | SF-36 | Usual Care | Resistance | NR | NR x 7 x 12 |
| (Fillion *et al.*, 2008) | 4 | Cancer | POMS | NT/WL | Aerobic | NR | 60 x 1 x 4 |
| (Galvão *et al.*, 2010) | 1 | Cancer | SF-36 | Usual Care | Aerobic + Resistance | Moderate | 35 x 2 x 12 |
| (García-Martínez, De Paz and Márquez, 2012) | 1 | Neuro | SF-36 | NT/WL | Aerobic + Resistance | Moderate | 55 x 3 x 12 |
| (Gavi *et al.*, 2014) | 1 | Neuro | SF-36 | Active | Resistance | Moderate | 45 x 2 x 16 |
| (Ghanem *et al.*, 2010) | 1 | Other | SF-36 | Usual Care | Aerobic + Resistance | NR | NR x 3.5 x 8 |
| (Hewett *et al.*, 2018) | 2 | Other | SF-36 | NT/WL | Yoga | NR | 90 x 4 x 16 |
| (Hoffman *et al.*, 2010) | 1 | Neuro | SF-36 | NT/WL | Aerobic | Moderate | 30 x 5 x 10 |
| (Hughes, Casal and Leon, 1986)* | 2 | Healthy | POMS | NT/WL | Aerobic | Moderate | 45 x 5 x 12 |
| (Kalron *et al.*, 2021) | 2 | Neuro | SF-36 | Active | Aerobic + Resistance | Moderate | 55 x 2 x 6 |
| (Kaltsatou *et al.*, 2014) | 2 | Heart | SF-36 | NT/WL | Aerobic + Resistance/Dance | Moderate | 60 x 3 x 32 |
| (Kargarfard *et al.*, 2012) | 2 | Neuro | Other | NT/WL | Aerobic | Moderate | 60 x 3 x 8 |
| (Kiecolt-Glaser *et al.*, 2014) | 2 | Cancer | SF-36 | NT/WL | Yoga | NR | 90 x 2 x 12 |
| (Kim *et al.*, 2010) | 1 | Cancer | SF-36 | Usual Care | Resistance | Moderate | 15 x 5 x 8 |
| (Kline *et al.*, 2012) | 3 | Other | POMS/SF-36 | Active | Aerobic + Resistance | Moderate | 60 x 4 x 12 |
| (Laredo-Aguilera *et al.*, 2018) | 2 | Healthy | POMS | NT/WL | Aerobic + Resistance | Moderate | 60 x 3 x 10 |
| (Mannerkorpi *et al.*, 2000)* | 1 | Neuro | SF-36 | NT/WL | Aerobic | NR | 35 x 1 x 24 |
| (Martin *et al.*, 2009) | 3 | Healthy | SF-36 | NT/WL | Aerobic | All | NR x 3.5 x 24 |
| (Martins *et al.*, 2011) | 4 | Healthy | POMS | NT/WL | Aerobic/Resistance | Moderate | 45 x 3 x 16 |
| (Mostert and Kesselring, 2002)* | 2 | Neuro/  Healthy | SF-36 | NT/WL | Aerobic | Moderate | 30 x 5 x 3.5 |
| (O’Connor *et al.*, 2018) | 3 | Other | POMS/SF-36 | Usual Care | Resistance | Moderate | 17 x 2 z 12 |
| (Oken *et al.*, 2004)* | 2 | Neuro | SF-36 | Usual Care | Aerobic/Yoga | Light | 90 x 1 x 24 |
| (Vos *et al.*, 2015) | 2 | Cancer | SF-36 | Active | Aerobic + Resistance | Moderate | 70 x 3 x 36 |
| (Petajan *et al.*, 1996) | 6 | Neuro | POMS | NT/WL | Aerobic | Moderate | 45 x 3 x 15 |
| (Peters *et al.*, 2002)* | 2 | Other | SF-36 | Active | Aerobic | Moderate | 60 x 2 x 10 |
| (Porserud, Sherif and Tollbäck, 2014) | 2 | Cancer | SF-36 | Usual Care | Aerobic + Resistance | NR | 45 x 2 x 12 |
| (Puetz, Flowers and O’Connor, 2008) | 24 | Other | POMS | NT/WL | Aerobic | Light/  Moderate | 20 x 3 x 6 |
| (Quittan *et al.*, 1999)* | 1 | Heart | SF-36 | NT/WL | Aerobic | Moderate | 60 x 2.5 x 12 |
| (Reid *et al.*, 2010) | 1 | Other | SF-36 | Active | Aerobic | Moderate | 25 x 4 x 16 |
| (Romberg, Virtanen and Ruutiainen, 2005) | 1 | Neuro | Other | NT/WL | Aerobic + Resistance | NR | NR x 4.5 x 26 |
| (Rossi *et al.*, 2014) | 1 | Other | SF-36 | NT/WL | Aerobic + Resistance | Moderate | 60 x 2 x 12 |
| (Sanudo *et al.*, 2010) | 2 | Neuro | SF-36 | NT/WL | Aerobic/Aerobic + Resistance | Moderate | 52.5 x 2 x 24 |
| (Segal *et al.*, 2001)* | 2 | Cancer | SF-36 | Usual Care | Aerobic | Moderate | NR x 5 x 26 |
| (Seki *et al.*, 2003)* | 1 | Heart | SF-36 | Usual Care | Aerobic + Resistance | Moderate | 65 x 3 x 24 |
| (Sillanpää *et al.*, 2012) | 3 | Healthy | SF-36 | NT/WL | Aerobic/Aerobic + Resistance/Resistance | Moderate | 65 x 2.7 x 21 |
| (Singh, Clements and Fiatarone, 1997)* | 1 | Other | SF-36 | Usual Care | Resistance | High | 50 x 3 x 10 |
| (Smith *et al.*, 2007) | 2 | Other | SF-36 | Active | Yoga | NR | 60 x 1 x 10 |
| (Smith *et al.*, 2017) | 1 | Healthy | SF-36 | NT/WL | Resistance | NR | 30 x 3 x 8 |
| (Sutherland, Andersen and Stoové, 2001)* | 3 | Neuro | POMS/Other | NT/WL | Aerobic + Resistance | NR | 45 x 3 x 10 |
| (Taaffe *et al.*, 2017) | 4 | Cancer | SF-36 | Usual Care | Aerobic + Resistance | Moderate | 30 x 2 x 48 |
| (Teixeira *et al.*, 2008) | 2 | Healthy | POMS | NT/WL | Other | Moderate | 50 x 3 x 19 |
| (Theander *et al.*, 2009) | 1 | Other | SF-36 | NT/WL | Aerobic + Resistance | NR | 60 x 2 x 12 |
| (Tomas-Carus *et al.*, 2009) | 1 | Neuro | SF-36 | NT/WL | Aerobic + Resistance | Moderate | 60 x 3 x 32 |
| (Travier *et al.*, 2015) | 1 | Cancer | SF-36 | Usual Care | Aerobic + Resistance | Moderate | 60 x 2 x 18 |
| (Tsai *et al.*, 2004)* | 2 | Heart | SF-36 | NT/WL | Aerobic | Moderate | 50 x 3 x 10 |
| (Tsutsumi *et al.*, 1998)* | 2 | Healthy | POMS | NT/WL | Resistance | Moderate/High | NR x 3 x 12 |
| (Van Vulpen *et al.*, 2016) | 1 | Cancer | SF-36 | Usual Care | Aerobic + Resistance | Moderate | 60 x 2 x 18 |
| (Wenzel *et al.*, 2013) | 2 | Cancer | POMS | Usual Care | Aerobic | Moderate | 60 x 5 x 20 |
| (Winters-Stone *et al.*, 2015) | 1 | Cancer | SF-36 | Active | Resistance | NR | 60 x 3 x 52 |
| (Winters-Stone *et al.*, 2016) | 6 | Cancer/  Healthy | SF-36 | Usual Care | Resistance | Moderate | 60 x 2 x 24 |
| (Yeo *et al.*, 2012) | 1 | Cancer | SF-36 | Usual Care | Aerobic | NR | 30 x 4 x 12 |
| (Yu *et al.*, 2003)* | 3 | Heart | SF-36 | NT/WL | Aerobic | Moderate | 120 x 2 x 34 |
| (Yu *et al.*, 2004) | 3 | Heart | SF-36 | Usual Care | Aerobic | Moderate | 120 x 2 x 34 |
| (Zanuso *et al.*, 2012) | 2 | Healthy | POMS | NT/WL | Resistance | Moderate | 60 x 3 x 12 |
| (Zhou *et al.*, 2017) | 1 | Cancer | SF-36 | Usual Care | Aerobic | Moderate | 30 x 5 x 24 |

Neuro = neurological sample; NR = not reported; NT/WL = no treatment/wait-list.
*Denotes studies included in this meta-analysis and our previous meta-analysis (Puetz, O’Connor and Dishman, 2006).
**Two articles on the same study.
