# Supplementary figures and images for: The Effect of Chronic Exercise on Energy and Fatigue States: A Systematic Review and Meta-Analysis of Randomized Trials
Source: Front Psychol. 2022 Jun 3;13:907637. doi: 10.3389/fpsyg.2022.907637 (PMC9206544; doi:10.3389/fpsyg.2022.907637)

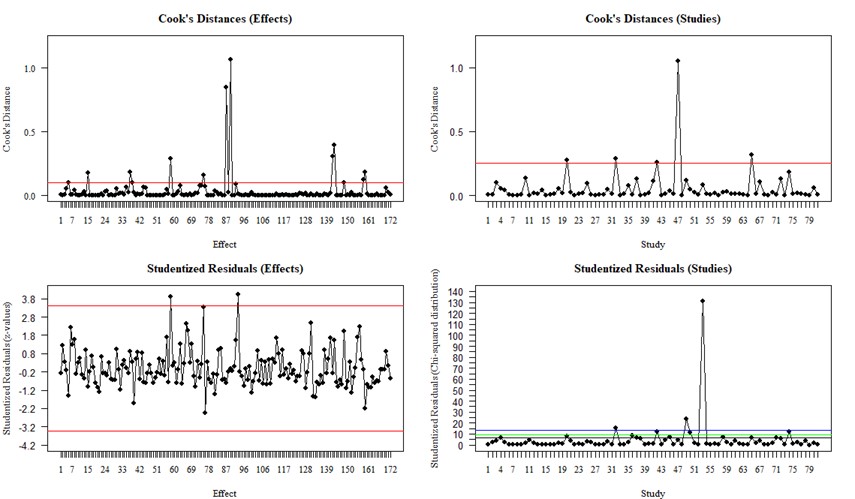

Supplement: Supplementary Figure 1 — Influential and outlier effects and studies. Cook’s distances and studentized residuals for effects and studies. For Cook’s distances, values exceeding the median plus six times the interquartile range are considered influential. For studentized residuals (effects), cut-off z-value is set at 3.44. For studentized residuals for studies, three Chi-squared critical values are displayed [black line, studies with one effect (z = 6.63); green line, studies with two effects (z = 9.21); blue line, studies with three effects (z = 13.28)]. Influential effects: Cramer et al. (1991) – vitality, Brown et al. (2001) – vitality, Sutherland et al. (2001) – fatigue and vitality, Teixeira et al., (2008) – energy, Ghanem et al. (2010) – vitality, Wenzel et al. (2013) – energy and fatigue, Laredo-Aguilera et al. (2018) – energy, O’Connor et al. (2018) – energy and vitality, and Araújo et al. (2021) – fatigue. Influential studies: Cramer et al. (1991), Sutherland et al. (2001), Ghanem et al. (2010), Laredo-Aguilera et al. (2018), and O’Connor et al. (2018). Outlier effects: Ghanem et al. (2010) – vitality and Paulo et al. (2019) – vitality. Outlier studies: Puetz et al. (2008), Ghanem et al. (2010), Wenzel et al. (2013), Laredo-Aguilera et al. (2018), and Paulo et al. (2019). [file Image_1.JPEG]
